# Modifying nutritional substrates induces macrovesicular lipid droplet accumulation and metabolic alterations in a cellular model of hepatic steatosis

**DOI:** 10.14814/phy2.14482

**Published:** 2020-07-08

**Authors:** Pippa J. Gunn, Camilla Pramfalk, Val Millar, Thomas Cornfield, Matthew Hutchinson, Elspeth M. Johnson, Shilpa R. Nagarajan, Perla Troncoso‐Rey, Richard F. Mithen, Katherine E. Pinnick, Maria H. Traka, Charlotte J. Green, Leanne Hodson

**Affiliations:** ^1^ Oxford Centre for Diabetes, Endocrinology and Metabolism Radcliffe Department of Medicine University of Oxford Oxford UK; ^2^ Division of Clinical Chemistry Department of Laboratory Medicine Karolinska Institutet at Karolinska University Hospital Huddinge Stockholm Sweden; ^3^ Target Discovery Institute Nuffield Department of Medicine University of Oxford Oxford UK; ^4^ Quadram Institute Bioscience Norwich Research Park UK; ^5^ National Institute for Health Research Oxford Biomedical Research Centre Oxford University Hospital Trusts Oxford UK

**Keywords:** fatty acid, lipid droplet, macrovesicular steatosis, metabolism, NAFLD, RNA sequencing, senescence

## Abstract

**Background and Aims:**

Nonalcoholic fatty liver disease (NAFLD) begins with steatosis, where a mixed macrovesicular pattern of large and small lipid droplets (LDs) develops. Since in vitro models recapitulating this are limited, the aims of this study were to develop mixed macrovesicular steatosis in immortalized hepatocytes and investigate effects on intracellular metabolism by altering nutritional substrates.

**Methods:**

Huh7 cells were cultured in 11 mM glucose and 2% human serum (HS) for 7 days before additional sugars and fatty acids (FAs), either with 200 µM FAs (low fat low sugar; LFLS), 5.5 mM fructose + 200 µM FAs (low fat high sugar; LFHS), or 5.5 mM fructose + 800 µM FAs (high fat high sugar; HFHS), were added for 7 days. FA metabolism, lipid droplet characteristics, and transcriptomic signatures were investigated.

**Results:**

Between the LFLS and LFHS conditions, there were few notable differences. In the HFHS condition, intracellular triacylglycerol (TAG) was increased and the LD pattern and distribution was similar to that found in primary steatotic hepatocytes. HFHS‐treated cells had lower levels of de novo‐derived FAs and secreted larger, TAG‐rich lipoprotein particles. RNA sequencing and gene set enrichment analysis showed changes in several pathways including those involved in metabolism and cell cycle.

**Conclusions:**

Repeated doses of HFHS treatment resulted in a cellular model of NAFLD with a mixed macrovesicular LD pattern and metabolic dysfunction. Since these nutrients have been implicated in the development of NAFLD in humans, the model provides a good physiological basis for studying NAFLD development or regression in vitro.

## Graphical abstract

Exposing Huh7 cells to repeated doses of a physiological treatment media containing, human serum, fatty acids and sugar lead to the development of a mixed macrovesicular steatotic lipid droplet pattern that is similar to that found in isolated primary hepatocytes. This model displays several characteristics of early‐stage NAFLD in vivo therefore providing a strong basis of a physiological model that can be further manipulated to investigate in vitro intracellular TAG development and/or regression.

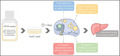

## 1 INTRODUCTION

Nonalcoholic fatty liver disease (NAFLD) has become a major public health burden (Younossi et al., 2016). NAFLD covers a spectrum of diseases, which starts with the accumulation of lipid droplets (LDs) within hepatocytes (steatosis). LDs typically have a triacylglycerol (TAG)‐rich core and were traditionally thought to have an inert role in energy metabolism; however, they are now recognized to be involved in diverse cellular processes (Welte & Gould, 2017). In hepatic steatosis, LDs may be mostly small, with poorly defined edges (microvesicular steatosis) or larger, distinct organelles (macrovesicular steatosis) (Takahashi & Fukusato, 2014); the latter can be further classified into small or large‐droplet macrosteatosis, depending on whether the LD present is large enough to displace the nucleus (Yersiz et al., 2013). Typically, macrovesicular steatosis is observed in the liver biopsies from NAFLD patients, although microvesicular steatosis is present in around 10%–15% of cases (Masterton, Ngoh, Lockman, Hayes, & Plevris, 2011; Takahashi & Fukusato, 2014; Tandra et al., 2011). It remains to be elucidated how these two LD patterns influence disease pathogenesis as evidence is limited and findings inconsistent (Masterton et al., 2011; Tandra et al., 2011).

In studying the mechanistic processes involved in LD accumulation and implications on the pathogenesis of NAFLD, available models have limitations. Investigating hepatic LDs in humans is challenging and relies on liver biopsies, meaning only cross‐sectional data can be obtained, while LD patterns in in vitro models are poorly defined. Although nutritional composition of media can be used successfully to induce the cellular stress associated with NAFLD in vitro (Breher‐Esch, Sahini, Trincone, Wallstab, & Borlak, 2018; Chavez‐Tapia, Rosso, & Tiribelli, 2012; Cui, Chen, & Hu, 2010; Kostrzewski et al., 2017; Kozyra et al., 2018; Lyall et al., 2018; Sahini & Borlak, 2016; Zhao et al., 2016), whether it is a micro‐ or macrovesicular LD pattern that develops is rarely reported. In addition, increased intrahepatocellular storage of TAG in steatosis can be associated with perturbed systemic and hepatocellular metabolism, including elevated delivery or synthesis of fatty acids (FAs) and reduced secretion or oxidation of FAs (Diraison, Moulin, & Beylot, 2003; Fabbrini et al., 2008; Green, Pramfalk, Morten, & Hodson, 2015; Lambert, Ramos–Roman, Browning, & Parks, 2014). As a result, studying change in metabolic flux in vitro is also relevant; however, where large LDs have been confirmed, cellular metabolism and function have not been investigated (Nativ et al., 2013; Pawella et al., 2014).

Given the lack of reported data on intracellular TAG accumulation in combination with LD pattern and hepatocellular FA metabolism in vitro the aims of this study were firstly, to create a cellular model of intrahepatocellular TAG accumulation with a LD pattern that reflects human NAFLD by manipulating nutritional substrates and secondly, to investigate intracellular metabolism under these conditions.

## 2 METHODS

### 2.1 Materials

All cell culture reagents were obtained from Life Technologies (Paisley, UK) unless otherwise stated. Fetal bovine serum (FBS) and human serum (HS) were purchased from Seralab (Haywards Heath, UK). Deuterated water (^2^H_2_O) was purchased from CK Isotopes (Ibstock, UK) and gas chromatography (GC) standards were from Sigma–Aldrich (Gillingham, UK). Deuterated oleate and palmitate were from Cambridge Isotope Laboratories (MA, USA). Fluorescent stains (BODIPY^®^ 493/503, and Hoescht 33342 Solution) were from Thermo Fisher Scientific (Hemel Hempstead, UK).

### 2.2 Cell culture

Human hepatocellular carcinoma cells (Huh7) were a gift from Dr Camilla Pramfalk (Karolinska Institutet, originally from Creative Dynamics Inc. NY, US). Cells were maintained at 37°C in 5% carbon dioxide in low‐glucose (5.5 mM) Dulbecco's modified Eagle's medium (DMEM) + Glutamax™ with 10% FBS, 1% nonessential amino acids (NEAAs), and 10,000 U/ml penicillin–streptomycin. Mycoplasma testing was carried out on regular basis with no contamination reported.

### 2.3 Experimental treatments

After passaging, cells were seeded and grown to confluence in maintenance media, before being cultured in HS‐containing media for 7 days. HS media consisted of phenol red‐free DMEM, 11 mmol/L glucose, 1% NEAAs, 10,000 U/mL penicillin–streptomycin, 1% sodium pyruvate, and 1% Glutamax™, with 2% HS. After this, cells were treated with one of three experimental treatments. All treatments consisted of HS media described above with 0.5 nM insulin and a combination of supplementary FAs and fructose. Low‐fat low‐sugar (LFLS) media had an additional 200 μM FAs, low‐fat high‐sugar (LFHS) media had 200 μM FAs and 5.5 mM fructose added, and high‐fat high‐sugar (HFHS) media had 800 μM FAs and 5.5 mM fructose added. FAs consisted of a physiological mix of oleic acid, palmitic acid, linolenic acid, and α‐linoleic acid at a ratio of 45:30:24:1. FAs were dissolved in ethanol before being conjugated to BSA at a stock concentration of 10 mM FAs to 10% (w/v) BSA and diluted to 800 μM or 200 μM before treatment. For measurement of FA oxidation, deuterated [D_31_]palmitate and [D_33_]oleate were used. For assessment of de novo lipogenesis (DNL), 10% heavy water (^2^H_2_O) was added to media and for all experiments, media was changed every 48–72 hr.

### 2.4 Isolation of primary hepatocytes

Liver tissue was obtained from patients undergoing surgery who had consented (NRES Committee South Central; Berkshire B 11/SC/0443) to the use of excess tissue (resection surplus) for research. Tissue from three livers that had 5, 25, and 35% simple steatosis, respectively, displaying mixed macrosteatosis, had primary hepatocytes isolated and cultured following the method of Green et al. (2017). Briefly, isolated cells were seeded into collagen‐coated plates at a density of 2 x 10^5^ cells per cm^2^ and incubated at 37°C for 3–4 hr in William's E buffer containing supplements (1% NEAAs, 1% GlutaMAX™, 2% HS, 100 nM dexamethasone, 100 nM insulin, and 0.375% FA‐free BSA). After this, media was changed to William's E maintenance media (11 mM glucose, 1% NEEA, 1% GlutaMAX™, 100 nM dexamethasone, 100 nM insulin, and 0.375% FA‐free BSA), and cells were cultured for 24 hr.

### 2.5 Western blotting, qRT‐PCR, and biochemical analysis

Western blotting, qRT‐PCR, and biochemical assays were carried out as previously described (Green, Johnson, et al., 2015; Gunn, Green, Pramfalk, & Hodson, 2017). For the biochemical analysis, intracellular and media concentrations of metabolites, including TAG and 3‐hydroxybutyrate (3‐OHB) were analyzed on the ILab 650 (Instrumentation Laboratory, Werfen; Warrington, UK) where assays had been optimized for low concentrations found in vitro and complete media was used for a background measurement and results normalized to protein concentration (Green, Johnson, et al., 2015; Gunn et al., 2017).

### 2.6 Gluconeogenesis assay

After treatment, cells were starved for 24 hr to ensure glycogen depletion. Cells were incubated with glucose‐ and serum‐free media with 20 mM sodium pyruvate for an hour. After this, the Glucose‐Glo™ Assay from Promega (WI, USA) was carried out on cell media according to manufacturer's instructions.

### 2.7 Lipoprotein separations

After treatment, media was aspirated and cells were washed three times PBS, before sitting in serum‐ and FA‐free DMEM for 10 min to ensure the removal of all lipoproteins from the HS. This last wash was collected and used as a background control, and the cells were cultured a further 24 hr in serum‐ and FA‐free DMEM. Media was collected and concentrated using Amicon Ultra‐15 centrifugal filter devices (Merck Millipore Ltd., Cork, Ireland), which were centrifuged at 4,500*g* for 20 min. Separation of lipoproteins in the cell media was achieved by size‐exclusion chromatography followed by online determination of cholesterol and TAG, as described previously (Parini, Johansson, Broijersen, Angelin, & Rudling, 2006). Samples were diluted 1:1,000 for apoB measurement using The Human apoB ELISA^PRO^ kit (Mabtach; Nacka, Sweden), which was carried out according to manufacturer's instructions; a Tecan plate reader was used to measure absorbance at 450 nm (Tecan Infinitite F200, Tecan Nordic AB; Stockholm, Sweden).

### 2.8 Determination of oxidative stress

To measure cellular oxidative stress, 10 μM 2′,7′‐dichlorofluorescin diacetate (DCF‐DA) was added to each well for 45 min. Fluorescence was red on an EnSpire^®^ Multimode Plate Reader (PerkinElmer; Beaconsfield, UK) at 485/535 nm. Hydrogen peroxide was measured using the ROS‐Glo™ H_2_O_2_ Assay (Promega; WI, USA), according to manufacturer's instructions. Luminescence was read on an EnSpire^®^ Multimode Plate Reader (PerkinElmer; Beaconsfield, UK).

### 2.9 Determination of cellular ATP production

Cellular ATP concentrations were determined using the CellTiter‐Glo Luminescent Cell Viability Assay (Promega; WI, USA) according to manufacturer's instructions. Luminescence was read on an EnSpire^®^ Multimode Plate Reader (PerkinElmer; Beaconsfield, UK). Hoechst staining to quantify cell number was carried out as detailed in Section 2.6; ATP concentrations were corrected to Hoescht fluorescence and are reported as arbitrary units.

### 2.10 Preparation of cells for INCell analysis

Cells were plated into a 96‐well, black‐walled, collagen‐coated plate with optically clear well bases under normal seeding procedures. After treatment, media was removed from wells and 400 nM MitoTracker Red, 10 μg/mL BODIPY and 1 μg/mL Hoechst 33342 was added to stain mitochondria, lipid droplets, and nuclei, respectively. Paraformaldehyde diluted to 4% was then added and cells were fixed and covered with PBS and refrigerated until imaging.

Plates were imaged on the INCell Analyzer 6000 (GE Healthcare; IL, USA). Images were taken from five planes and nine fields per well, and four technical replicates were analyzed per passage. A Nikon 40×/0.60 objective was used to capture nine fields per well through five *Z*‐slices with an excitation and emission of 405/455 nm for Hoechst and 488/525 nm for BODIPY. Laser autofocus was used and optimal exposure settings and focus offsets were determined for each channel.

Images obtained were processed by the INCell Investigator‐Developer toolbox 1.9.2 (GE Healthcare; IL, USA). Images from the FITC channel were used to analyze LDs, which were first identified using a preprocessing algorithm and segmented based on their fluorescence intensity. A postprocessing algorithm applying erosion, clump breaking and a spherical form factor was used to separate groups of LDs into individual targets for quantification; several targets were checked manually before data export.

### 2.11 Lipid extraction and gas chromatography–mass spectrometry

Lipid was extracted from cell lysate and media according to the Folch method (Folch, Lees, & Sloane Stanley, 1957) and prepared and analyzed by a 6890N Network GC System (Agilent Technologies; CA, USA) as previously described (Burdge, Wright, Jones, & Wootton, 2000; Gunn et al., 2017). Fatty acid methyl esters were identified by their retention times compared to a standard containing 31 known FAs and quantified. The micromolar quantities were then totalled and each FA was expressed as a percentage of this value (molar percentage; mol%).

Intracellular DNL was assessed based on the incorporation of deuterium from ^2^H_2_O in the media (Finnigan GasBench‐II, ThermoFisher Scientific, UK) into [^2^H]FAs in intracellular TAG and PL and media TAG; the presence of [^2^H]FAs indicates that DNL from nonlipid precursors has synthesis these FAs. [^2^H]palmitate, [^2^H]palmitoleate, [^2^H]stearate, and [^2^H]oleate enrichments were determined simultaneously by GC‐mass spectrometry (GC‐MS) using a 5890 GC coupled to a 5973N MSD (Agilent Technologies; CA, USA). Ions with mass‐to‐charge ratios (m/z) of M + 0 and M + 1 were determined by selected ion monitoring (Semple et al., 2009). As a marker of FA oxidation, we measured the appearance of ^2^H_2_O using a Finnigan GasBench‐II (ThermoFisher Scientific, UK) in cell media derived from the [D_31_]palmitate and [D_33_]oleate in the media (Law et al., 2007).

### 2.12 RNA extraction and RNA sequencing

RNA was extracted using the RNEasy^®^ Plus mini kit (QIAGEN Sciences; MD, USA) according to manufacturer's instructions. The resulting RNA was treated with DNase (QIAGEN Sciences; MD, USA) and quality checked with an Agilent Bioanalyzer.

Samples with RIN values higher than 7 (24 out of 28 samples) were used for library preparation using the NEB Next^®^ Ultra RNA Library Prep Kit. Sequencing of 24 libraries was performed on Illumina's NovaSeq 6000 to produce 100 bp paired‐end reads, generating 30 million reads per library. RNA‐sequencing reads were first processed by removing Illumina adaptors, low‐quality reads (Phred score < 30) and short reads (<60 bp), using *Trim Galore!* version 0.4.2 (Babraham Bioinformatics). SortMeRNA version 2.1 (Kopylova, Noe, & Touzet, 2012) was used to filter any remaining ribosomal RNA from the adaptor and quality‐trimmed reads. High‐quality reads were then processed to generate raw gene counts following the *HISAT2* and *StringTie* protocol (Kim, Langmead, & Salzberg, 2015). Reads were aligned to the reference genome (ensembl GRCh38.89 reference genome) using *HISAT2* (version 2.0.5). Alignments were then assembled into full length transcripts and quantified using *StringTie* (version 1.3.3) and gene counts were exported into *edgeR* in *R* Bioconductor (Robinson, McCarthy, & Smyth, 2010).

RNA sequencing data from the study have been deposited in the ArrayExpress database at EMBL‐EBI (www.ebi.ac.uk/arrayexpress) under the accession number E‐MTAB‐7884.

### 2.13 Differential gene expression analyses

Differential gene expression (DGE) analysis following calculation of normalized gene counts was undertaken in *R* using *limma* and *voom* transformation (Ritchie et al., 2015).

### 2.14 Functional analyses

Functional analyses of paired DGE was undertaken by the gene set enrichment analysis (GSEA) software (Subramanian et al., 2005) using gene sets derived from the Reactome pathway database (674 gene sets in total) within the available Molecular Signatures Database (v6.1 MSigDB). Differential gene expression lists were ranked according to their *p*‐value, modified using the rank–rank hypergeometric overlap (RRHO) algorithm (Plaisier, Taschereau, Wong, & Graeber, 2010).

Modified *p*‐values were calculated as the signed log10‐transformed *p*‐value of the log fold between different treatments, with the sign denoting the direction of the change; positive for upregulated and negative for downregulated. By using the RRHO method, we explored the functional consequences of the changes in gene expression without being constraint by a given statistical threshold. The ranked DGE list was then submitted to gene set enrichment analysis (GSEA).

### 2.15 Statistics

All experiments consisted of a minimum of three replicates and data are presented as mean ± *SEM*. GraphPad Prism 7 (GraphPad Software, Inc.; CA, USA) was used for data analysis. One‐ and two‐way ANOVAs with Bonferroni's multiple comparisons tests were used to determine significant differences (*p* < .05).

For DGE analysis of RNA‐sequencing data, adjustment for multiple‐testing was performed using the Benjamini–Hochberg false discovery rate (FDR) method to generate a *q*‐value. Functional analysis had statistical significance of enriched pathways set at an FDR‐adjusted *p* < .05. Normalized enrichment scores (NES) for each individual pathway and their associated FDR‐adjusted *p*‐value for each treatment were reported.

## 3 RESULTS

### 3.1 The effect of media composition on intracellular TAG content on lipid droplet pattern

**FIGURE 1:**
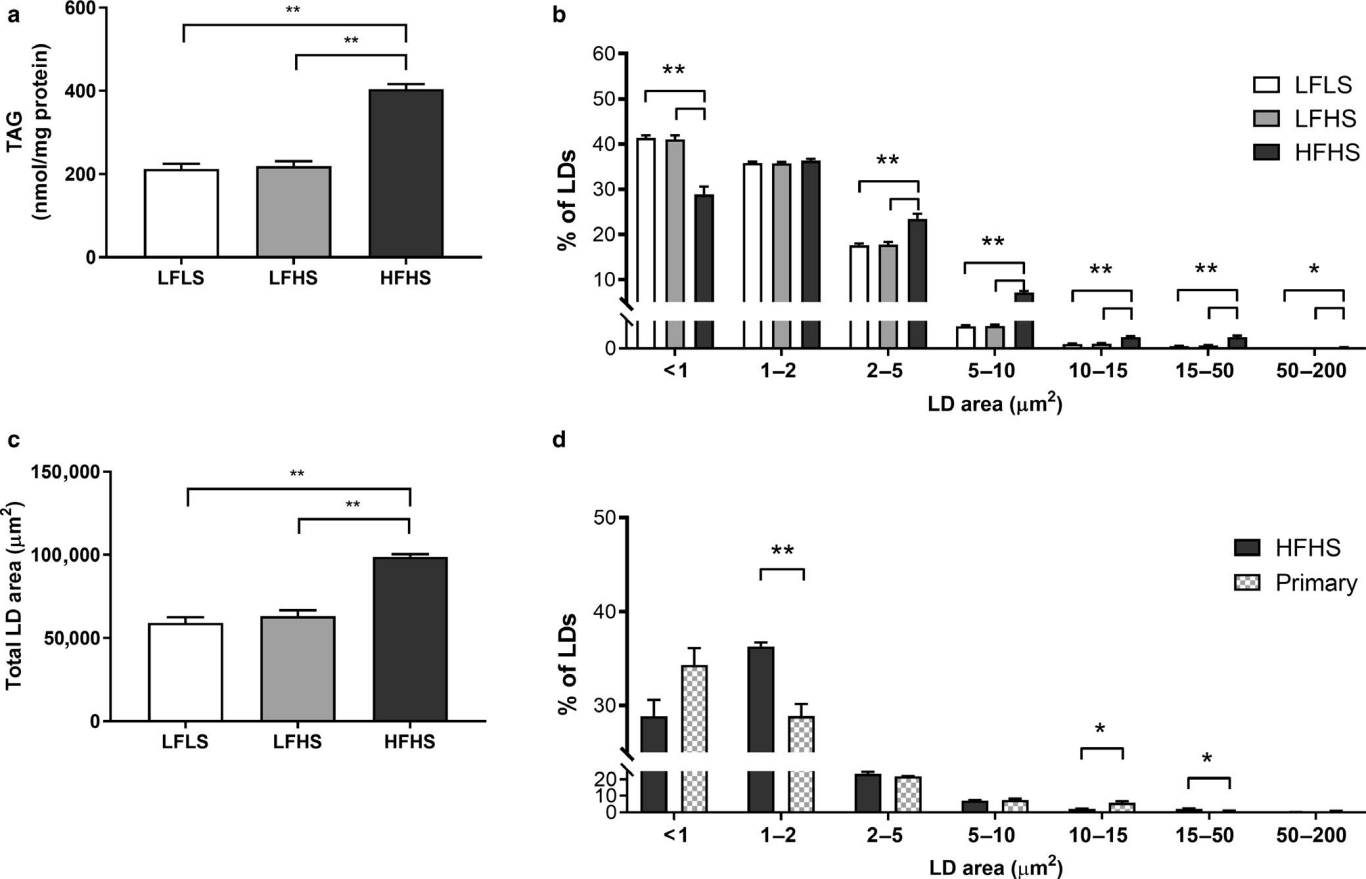
The effect of media composition on triacylglycerol (TAG) accumulation and lipid droplet (LD) characteristics. Once confluent, cells were treated with media containing 2% human serum and 11 mM glucose for 7 days before treatment media was added. Treatments consisted of either low fat low sugar (LFLS; 11 mM glucose + 200 μM FAs), low fat high sugar (LFHS; 200 μM FAs + 11 mM glucose + 5.5 mM fructose) or high fat high sugar (HFHS; 800 μM FAs + 11 mM glucose + 5.5 mM fructose); all treatments contained 0.5 nM insulin. (a) Intracellular triacylglycerol (TAG), (b) total LD area, and (c) distribution of LD area were measured; (d) primary hepatocytes isolated from liver tissue (one nonsteatotic and two steatotic) were cultured for 24 hr and LD populations were compared to HFHS‐treated Huh7 cells. Data are mean ± *SEM*, *n* = 6 for Huh7 cells and *n* = 3 for primary cells. *p < .05, **p < .001; two‐way ANOVA with Bonferroni's multiple comparisons test

**FIGURE 2:**
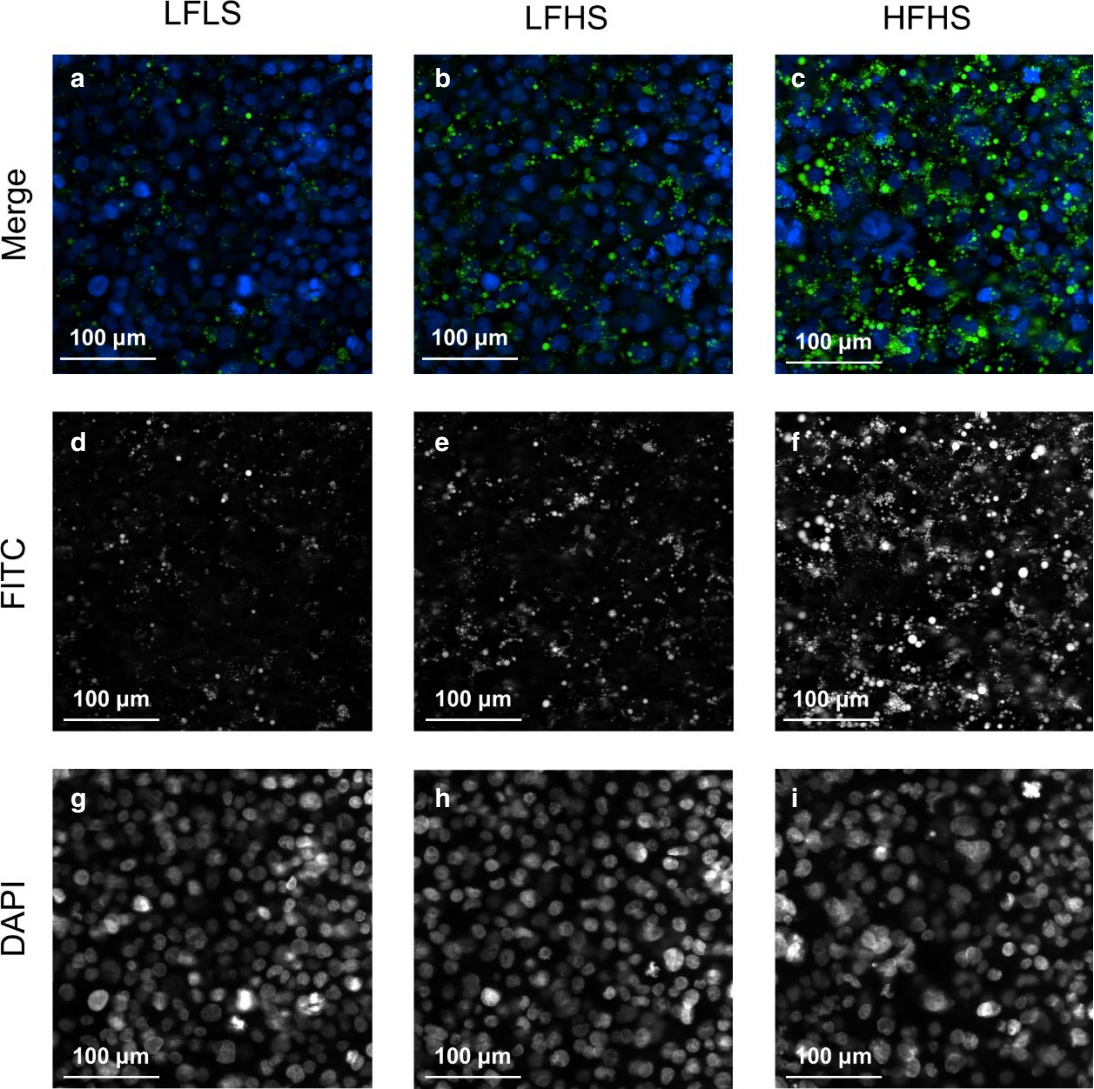
The effect of media composition on lipid droplet accumulation. Once confluent, cells were treated with media containing 2% human serum and 11 mM glucose for 7 days before treatment media was added. Treatments consisted of either low fat low sugar (LFLS; 11 mM glucose + 200 μM FAs), low fat high sugar (LFHS; 200 μM FAs + 11 mM glucose + 5.5 mM fructose) or high fat high sugar (HFHS; 800 μM FAs + 11 mM glucose + 5.5 mM fructose); all treatments contained 0.5 nM insulin. Cells were stained with Hoechst (nuclei; blue) and BODIPY (LDs; green) and representative fluorescence images were obtained at 40× magnification. Merged images (a–c) are comprised of signals from the FITC (d–f) and DAPI (g–i) channels to capture lipid droplets and nuclei, respectively

**FIGURE 3:**
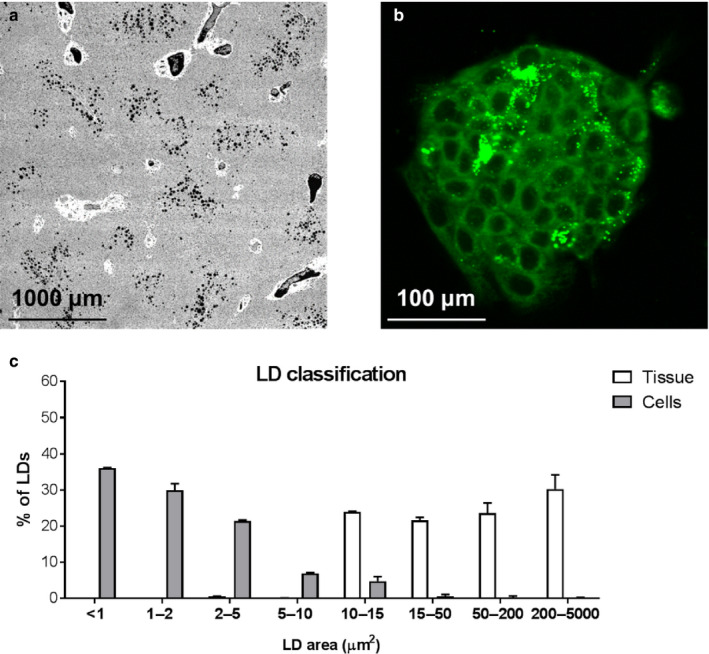
Differences in lipid droplet size distributions in whole tissue and isolated primary hepatocytes. Representative liver tissue slide FITC image at 4X magnification with lipid droplets shown in black (a). Representative image of BODIPY 493/503 staining in isolated hepatocytes (40× magnification; b). Frequency distribution of lipid droplet size in liver tissue and primary hepatocytes isolated from the same livers (c). Data are mean ± *SD*, *n* = 3

Culturing the cells in different media compositions resulted in intracellular TAG content being similar between the LFLS and LFHS conditions, but significantly (*p* < .01) lower compared to the HFHS‐treated cells (Figure 1a). To determine whether differences in intracellular TAG accumulation translated to differences in LD characteristics, LD size, and number were quantified from INCell imaging (Figure 2). Overall, there were significantly fewer LDs per cell in the LFLS‐treated (7.8 ± 0.5, mean ± *SEM*) compared to the LFHS‐ (9.3 ± 0.1) and HFHS‐treated (10.0 ± 0.1) cells (both *p* < .05); the total area of LDs was significantly higher in HFHS‐treated compared to LF‐treated cells (both *p* < .001; Figure 1b). Stratifying LDs by area revealed that cells treated with LFLS and LFHS media had significantly more small LDs (<1 μm^2^) and significantly fewer large LDs (2–200 μm^2^; Figure 1c) compared to HFHS‐treated cells; however, mRNA expression of LD proteins remained unaltered (Table 1). When compared to primary human cells isolated from steatotic liver tissue, the LD population from HFHS cells were comparable, although the primary cells had significantly more LDs between 10–50 μm^2^, and fewer small LDs (1–2 μm^2^) (Figures 1d and 3).

**TABLE 1:** The effect of media composition on gene expression in Huh7 cells

	LFLS	LFHS	HFHS
FA and TAG synthesis genes			
*ACACA*	1.08 ± 0.08^a^	1.02 ± 0.06^a^	0.72 ± 0.02^b^
*ACACB*	0.96 ± 0.04	0.89 ± 0.06	0.93 ± 0.04
*DGAT1*	0.91 ± 0.06	0.92 ± 0.07	0.79 ± 0.02
*DGAT2*	0.98 ± 0.06	1.09 ± 0.05	0.97 ± 0.05
*FASN*	0.88 ± 0.12	1.12 ± 0.17	0.87 ± 0.10
*SCD*	1.07 ± 0.06	1.31 ± 0.12	1.00 ± 0.10
FA elongation and desaturation genes			
*ELOVL2*	1.05 ± 0.04	0.98 ± 0.06	0.91 ± 0.04
*ELOVL5*	1.01 ± 0.01^a^	0.96 ± 0.03	0.89 ± 0.03^b^
*ELOVL6*	1.02 ± 0.09	1.05 ± 0.09	0.94 ± 0.05
*FADS1*	0.86 ± 0.03^a^	1.07 ± 0.06^b^	0.85 ± 0.05^a^
*FADS2*	0.97 ± 0.04	1.14 ± 0.06^a^	0.87 ± 0.04^b^
Lipid droplet protein genes			
*BSCL2*	0.79 ± 0.02	0.82 ± 0.04	0.80 ± 0.04
*CIDEB*	0.91 ± 0.11	1.06 ± 0.11	0.96 ± 0.07
*CIDEC*	0.91 ± 0.09	0.92 ± 0.11	0.99 ± 0.11
*PLIN1*	0.79 ± 0.05	0.98 ± 0.10	1.04 ± 0.09
*PLIN2*	0.95 ± 0.05	1.02 ± 0.05	0.99 ± 0.04
*PLIN3*	0.95 ± 0.04	1.06 ± 0.08	0.98 ± 0.07
*PLIN5*	0.78 ± 0.05	0.89 ± 0.05	0.92 ± 0.12
Hepatic nuclear receptors & transcription factors			
*MLXIPL*	0.95 ± 0.03	1.09 ± 0.08	0.97 ± 0.07
*NR1H3*	0.94 ± 0.03	1.07 ± 0.07	0.90 ± 0.06
*NR1H4*	0.93 ± 0.05	0.96 ± 0.06	0.91 ± 0.05
*SREBF1*	0.96 ± 0.06	1.15 ± 0.05^a^	0.81 ± 0.04^b^

Note

Data are mean ± *SEM*, *n* = 6. Different letters denote significant differences between means, *p* < .05; two‐way ANOVA with Bonferroni's multiple comparisons test.

### 3.2 The effect of media composition on intracellular DNL, FA desaturation, and elongation

**FIGURE 4:**
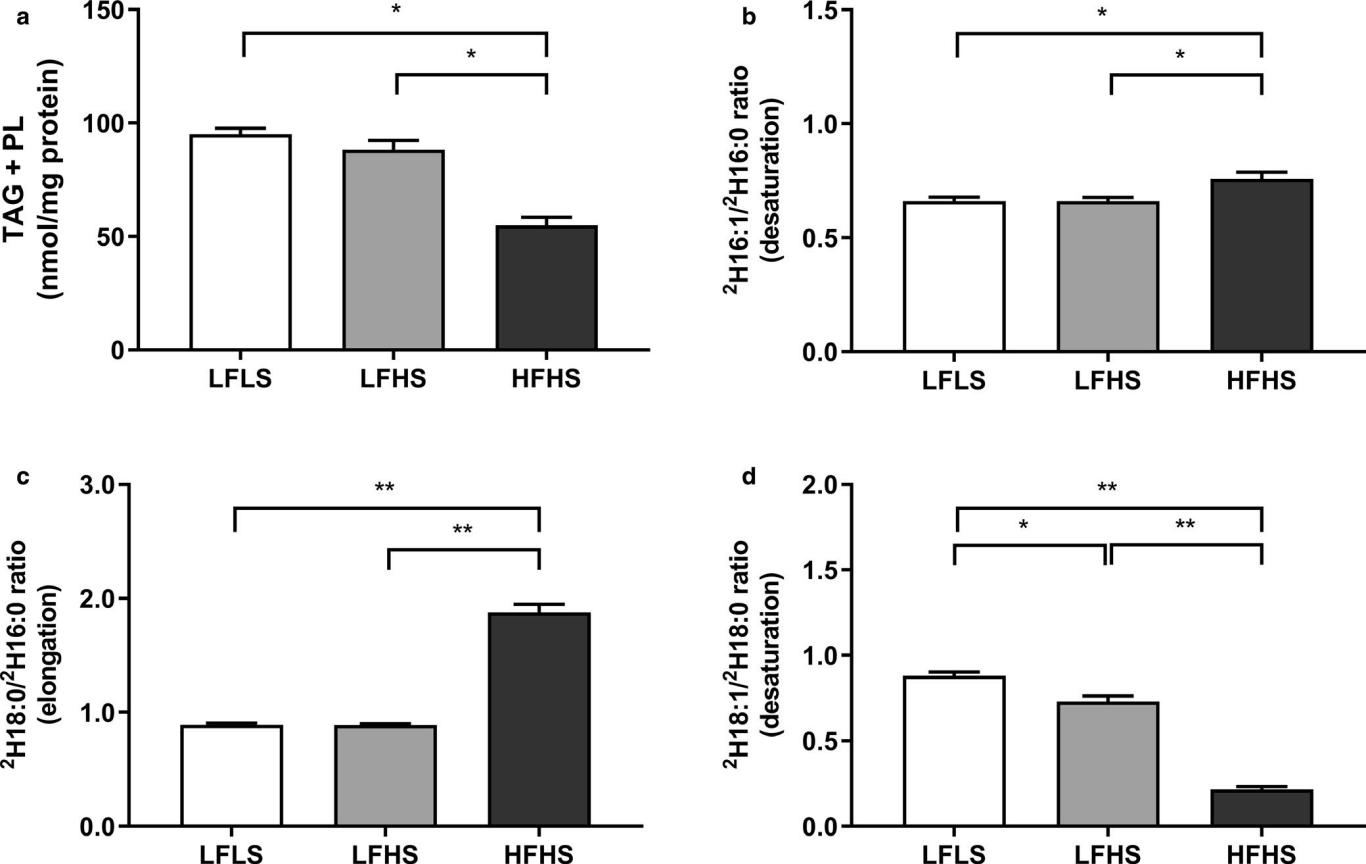
The effect of media composition on fatty acid (FA) synthesis and metabolism in Huh7 cells. Once confluent, cells were treated with media containing 2% human serum and 11 mM glucose for 7 days before treatment media was added. Treatments consisted of either low fat low sugar (LFLS; 11 mM glucose + 200 μM FAs), low fat high sugar (LFHS; 200 μM FAs + 11 mM glucose + 5.5 mM fructose) or high fat high sugar (HFHS; 800 μM FAs + 11 mM glucose + 5.5 mM fructose); all treatments contained 0.5 nM insulin. The contribution of de novo lipogenesis was calculated from deuterium appearance in C16:0, C16:1*n*−7, C18:0 and C18:1*n*−7 & 9 in (a) total FAs, and (b) indices of C16:0 desaturation ([^2^H]16:1/[^2^H]16:0) and (c) elongation ([^2^H]18:0/[^2^H]16:0), and (d) C18:0 desaturation ([^2^H]18:1/[^2^H]18:0) were calculated and results corrected to protein content. Data are mean ± *SEM*, *n* = 6. *p < .05; **p < .001; two‐way ANOVA with Bonferroni's multiple comparisons test

One source of intracellular FAs are those synthesized de novo; these may be partitioned into TAG or phospholipid (PL) pools. Rather than measuring only DNL‐derived 16:0, which is often considered the end product of DNL, we measured other FAs that could be derived from de novo synthesized C16:0, as done by Wilke et al. (2009). Despite differences in total intracellular TAG, there were not differences in the total amount of TAG derived from DNL (LFLS 18.8 ± 1.1, LFHS 17.0 ± 1.3, HFHS 18.0 ± 1.4 nmol/mg protein). However, there were differences in the amount of DNL‐derived PLs (LFLS 76.3 ± 2.3, LFHS 71.3 ± 3.1, HFHS 37.0 ± 2.6 nmol/mg protein; LFLS and LFHS vs. HFHS, *p* < .001). As a result, when TAG and PL fractions were combined, differences in total DNL‐derived FAs between treatments were evident: HFHS‐treated cells had a lower proportion of DNL‐derived FAs compared to LFLS and LFHS‐treated cells (both *p* < .05; Figure 4a). In line with this, the expression of sterol regulatory element binding protein 1 (SREBP1; *SREBF1)* and acetyl‐CoA carboxylase 1 (ACC1; *ACACA*) were significantly lower (*p* < .05) in cells treated with HFHS media compared to cells treated with LFHS media ‐Table 1). Taken together, these data demonstrate that the higher intracellular TAG content in the HFHS cells resulted from esterification of exogenous media FAs, rather than from DNL‐derived FAs.

De novo synthesized palmitate can be further metabolized (via desaturation and elongation pathways) to other FAs; therefore, the product to precursor isotopic ratios of all newly synthesized FAs for [^2^H]16:1/[^2^H]16:0 and [^2^H]18:1/[^2^H]18:0 and [^2^H]18:0/[^2^H]16:0 were used as surrogate markers of enzyme desaturation by stearoyl‐CoA desaturase (SCD) and elongation by the elongase ELOVL6, respectively. In the HFHS‐treated cells, both the isotopic desaturation ([^2^H]16:1/[^2^H]16:0) and elongation ([^2^H]18:0/[^2^H]16:0) indices were significantly higher (*p* < .05), while the [^2^H]18:1/[^2^H]18:0 index was significantly lower (*p* < .01) in the HFHS‐treated compared to LF‐treated cells, despite being also desaturated by SCD (Figure 4b–d).

### 3.3 The effect of media composition on FA composition

**FIGURE 5:**
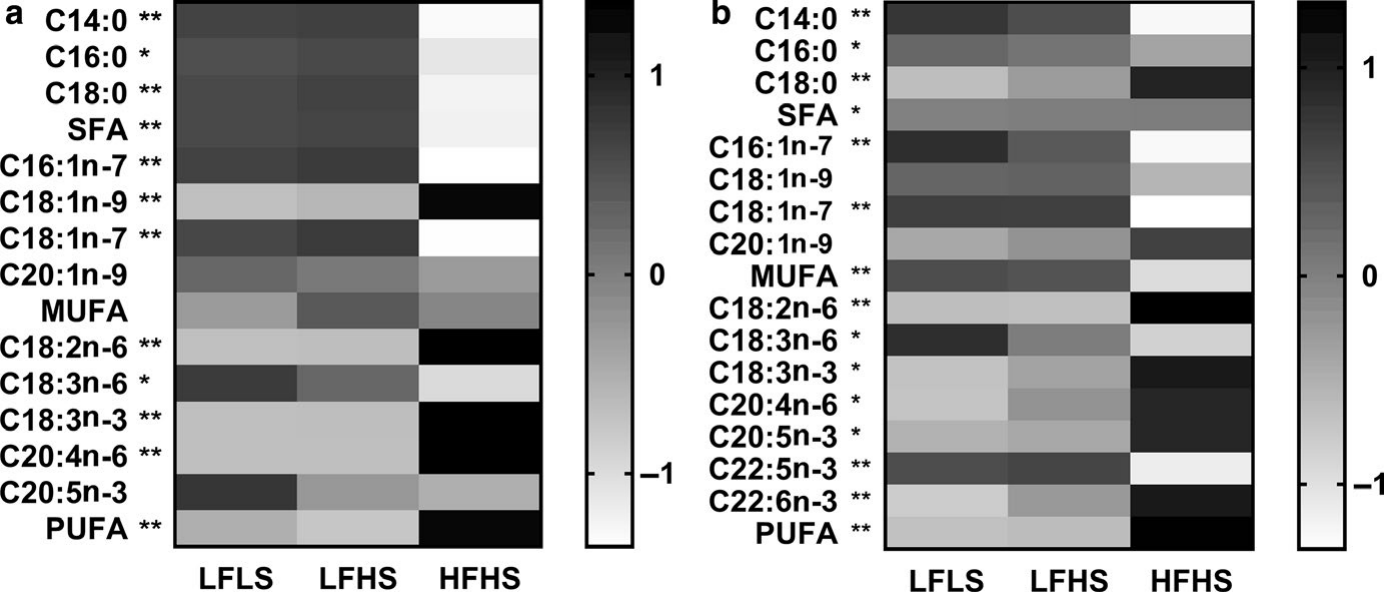
The effect of media composition on triacylglycerol and phospholipid fatty acid (FA) composition in Huh7 cells. Once confluent, cells were treated with media containing 2% human serum and 11 mM glucose for 7 days before treatment media was added. Treatments consisted of either low fat low sugar (LFLS; 11 mM glucose + 200 μM FAs), low fat high sugar (LFHS; 200 μM FAs + 11 mM glucose + 5.5 mM fructose) or high fat high sugar (HFHS; 800 μM FAs + 11 mM glucose + 5.5 mM fructose); all treatments contained 0.5 nM insulin. Fatty acids within (a) intracellular triacylglycerol (TAG) and (b) phospholipids (PL) were measured and corrected to protein content. Values are relative mol% contribution to total FAs (z‐scores), *n* = 6. *p < .05; **p < .001 LFLS and LFHS versus HFHS; two‐way ANOVA with Bonferroni's multiple comparisons test

Differences in the FA composition of intrahepatocellular TAG and PL have previously been reported in patients with and without NAFLD (Araya et al., 2004; Elizondo et al., 2007) and may play a key role in cellular function; therefore, we measured the intracellular FA composition of the cells. HFHS‐treated cells had a significantly lower proportion of intracellular saturated FAs in the TAG fraction and a significantly higher proportion of polyunsaturated FAs (*p* < .05; Figure 5a). Similar results were seen in the PL fraction, with HFHS‐treated cell PLs also having a lower proportion of monounsaturated FAs (*p* < .05; Figure 5b). Of the four exogenous FAs given, only palmitate (C16:0) was present at significantly lower proportions in HFHS‐treated compared to LF‐treated cells in both TAG and PL fractions, suggesting cells were particularly efficient at further metabolizing this FA, as evidenced by the increase in desaturation and elongation in these cells.

The total proportion of polyunsaturated FAs in TAG and PL were significantly higher in the HFHS cells compared to the LFLS and LFHS cells (Figure 5a and b). Notably, the PL *n*−6/*n*−3 ratio was higher in HFHS‐treated cells compared to LF‐treated cells (12.7 ± 0.25 vs. 8.89 ± 0.26 in LFLS and 8.70 ± 0.29 in LFHS, both *p* < .001). In line with these findings, gene expression in HFHS cells showed a significantly lower expression of the fatty acid desaturases *FADS1* and *2* compared to LFHS cells, and of *ELOVL5* compared to LFLS cells (all *p* < .05; Table 1).

### 3.4 The effect of media composition on lipoprotein secretion

**FIGURE 6:**
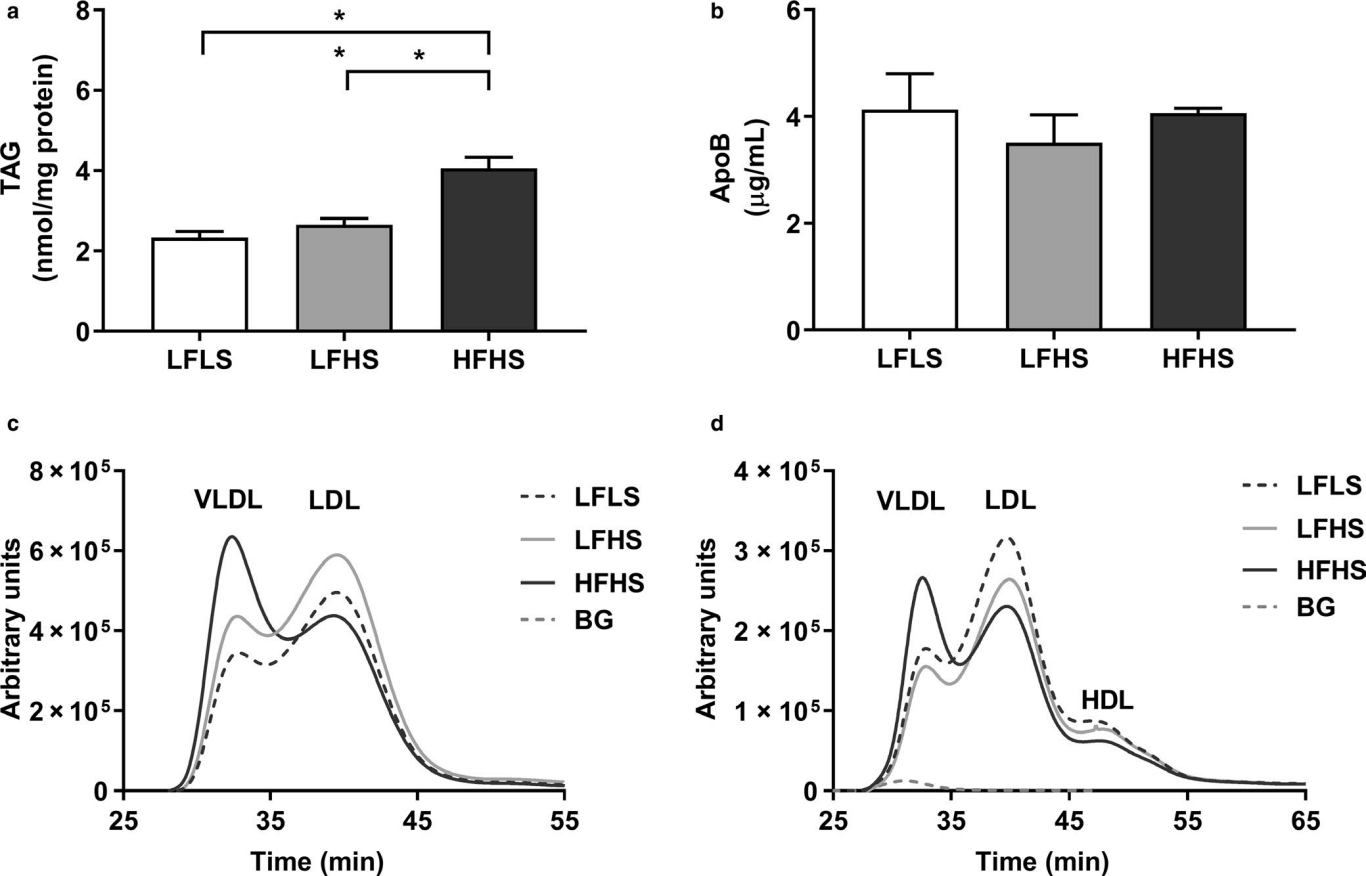
The effect of media composition on lipoprotein secretion. Once confluent, cells were treated with media containing 2% human serum and 11 mM glucose for 7 days before treatment media was added. Treatments consisted of either low fat low sugar (LFLS; 11 mM glucose + 200 μM fatty acids), low fat high sugar (LFHS; 200 μM fatty acids + 11 mM glucose + 5.5 mM fructose) or high fat high sugar (HFHS; 800 μM fatty acids + 11 mM glucose + 5.5 mM fructose); all treatments contained 0.5 nM insulin. (a) Media triacylglycerol (TAG) and (b) apoB were measured as markers of secretion. (c) Media TAG and (d) cholesterol content were measured in lipoproteins of different sizes by size exclusion chromatography. For media TAG, results are corrected to protein content and data are mean ± *SEM*, *n* = 6, for media apoB, *n* = 4. Lipoprotein curves are an average from two replicates; background (BG) measurement for TAG was below zero and is therefore not visible on the graph. *p < .05, **p < .001; two‐way ANOVA with Bonferroni's multiple comparisons test

Mobilized intrahepatocellular FA may be partitioned toward secretion as TAG in VLDL particles. There was a significantly higher media TAG concentration from HFHS‐treated cells compared to LFLS‐ and LFHS‐treated cells (*p* < .05; Figure 6a). Media ApoB was measured as a marker of particle number, which was not different between treatments (Figure 6b); indicating particles being secreted by the HFHS‐treated cells were more TAG‐enriched than LFLS‐ and LFHS‐treated cells. In line with this, size‐exclusion chromatography illustrated that HFHS‐treated cells were secreting VLDL‐sized particles, while the LFLS‐ and LFHS‐treated cells secreted more LDL‐sized particles (Figure 6c). Similar results were seen in cholesterol content, with most cholesterol secreted in VLDL, rather than LDL, sized particles in HFHS compared to low fat‐treated cells (Figure 6d). These lipoproteins appeared to be synthesized and secreted by the cells, since background measurements using the last wash from media that did not contain HS showed no detectable TAG and trace amounts of cholesterol (5.5 ± 0.3 nmol/L, compared to 275–300 nmol/L found in cell media).

### 3.5 The effect of media composition on ketogenesis, FA oxidation, ATP production, and oxidative stress

**FIGURE 7:**
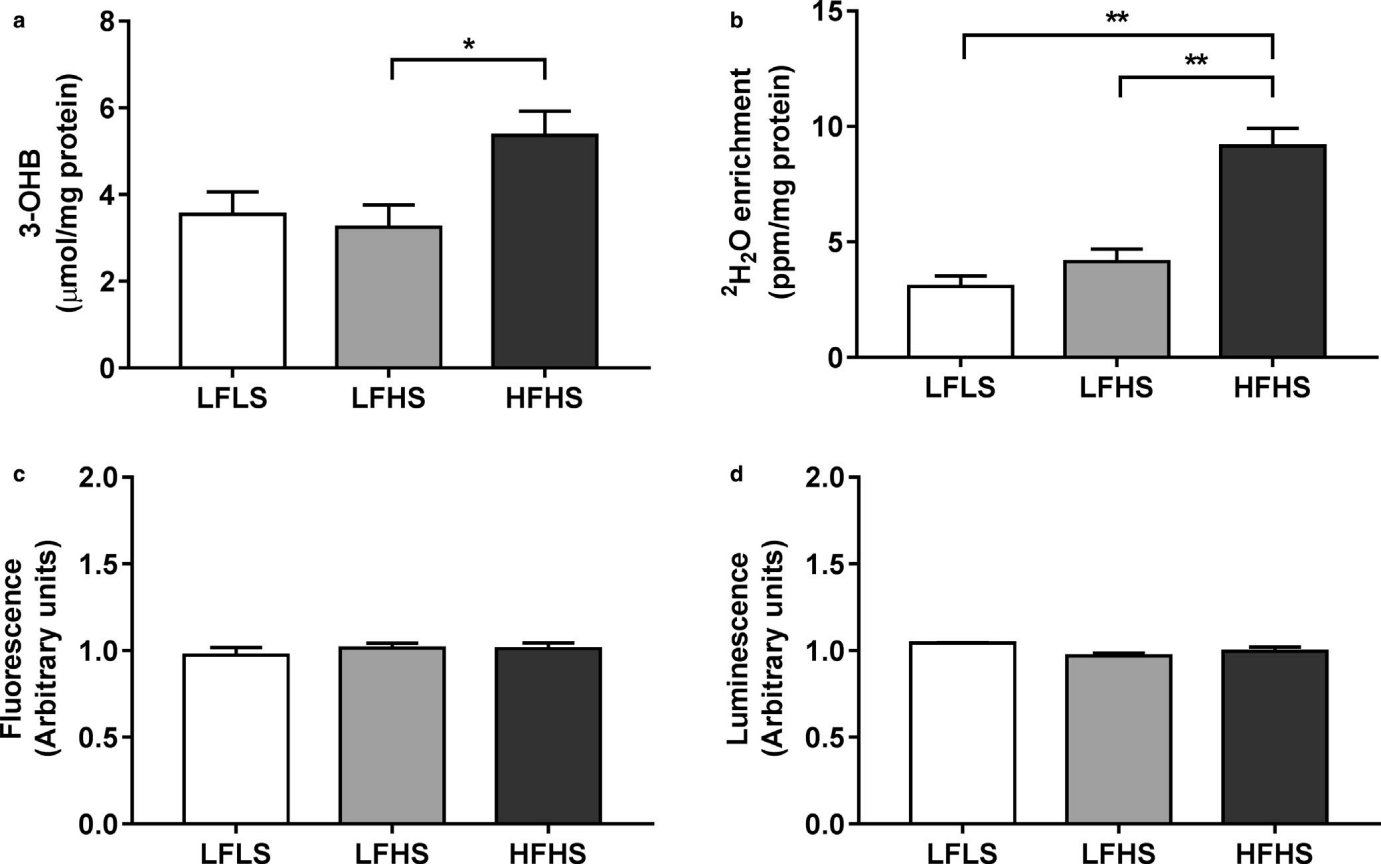
The effect of media composition on ketogenesis, fatty acid oxidation, and oxidative stress in Huh7 cells. Once confluent, cells were treated with media containing 2% human serum and 11 mM glucose for 7 days before treatment media was added. Treatments consisted of either low fat low sugar (LFLS; 11 mM glucose + 200 μM fatty acids), low fat high sugar (LFHS; 200 μM fatty acids + 11 mM glucose + 5.5 mM fructose) or high fat high sugar (HFHS; 800 μM fatty acids + 11 mM glucose + 5.5 mM fructose); all treatments contained 0.5 nM insulin. (a) Media 3‐hydroxybutyrate (3‐OHB), (b) media ^2^H_2_O enrichment, (c) fluorescence of 2',7'‐dichlorofluorescein (DCF), and (d) ROS‐Glo^TM^ luminesce were measured as markers of ketogenesis, fatty acid oxidation and oxidative stress, respectively. 3‐OHB and ^2^H_2_O results are corrected to protein content and ROS‐Glo^TM^ signal to Hoechst staining. Data are mean ± *SEM*, *n* = 4–6. *p < .05, **p < .001; two‐way ANOVA with Bonferroni's multiple comparisons test

Mobilized intrahepatocellular FA can also be partitioned into oxidation pathways, which may result in complete oxidation or ketogenesis. Media concentrations of the ketone body 3‐OHB were significantly higher in the HFHS condition compared to the LFHS condition (*p* < .05; Figure 7a). The production of ^2^H_2_O from [^2^H]FAs was used to measure complete oxidation through the electron transport chain; this was also significantly elevated in HFHS‐treated cells (*p* < .001; Figure 7b). ATP levels were not different between groups (LFLS 0.13 ± 0.04, LFHS 0.08 ± 0.02, HFHS 0.10 ± 0.03 arbitrary units; all *p* = *NS*). Elevated oxidative activity may result in oxidative stress within the cell; however, there was no significant difference in the cellular 2',7'‐dichlorofluorescein (DCF) fluorescence between treatments (Figure 7c), which was confirmed with measurement of H_2_O_2_ *via* the ROS‐Glo™ H₂O₂ Assay (Figure 7d).

### 3.6 The effect of media composition on glucose metabolism

**FIGURE 8:**
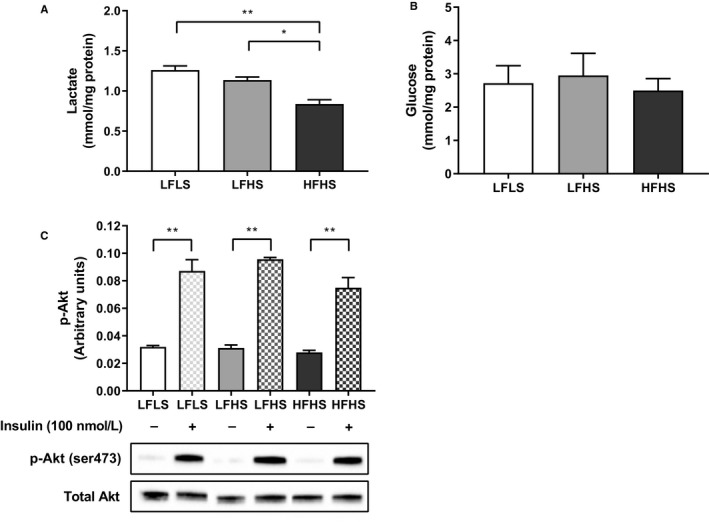
The effect of media composition on glucose metabolism and insulin signaling in Huh7 cells. Once confluent, cells were treated with media containing 2% human serum and 11 mM glucose for 7 days before treatment media was added. Treatments consisted of either low fat low sugar (LFLS; 11 mM glucose + 200 μM fatty acids), low fat high sugar (LFHS; 200 μM fatty acids + 11 mM glucose + 5.5 mM fructose) or high fat high sugar (HFHS; 800 μM fatty acids + 11 mM glucose + 5.5 mM fructose); all treatments contained 0.5 nM insulin. (a) Media lactate was measured as a marker of anaerobic glycolysis, results are corrected to protein content (lactate); *n* = 6. (b) Glucose remaining in the media after 24 hr in culture was measured. (c) Phosphorylation of Akt (ser473) was measured by ELISA and confirmed with Western blotting; *n* = 3. Data are mean ± *SEM*, *p < .05, **p < .001; two‐way ANOVA with Bonferroni's multiple comparisons test

The liver has metabolic flexibility in substrate use, with changes in FA metabolism having the potential to alter glucose metabolism. HFHS‐treated cells had significantly less media lactate (all *p* < .05; Figure 8a), suggesting that the provision of additional FAs diverted glucose away from anaerobic glycolysis. This was not due to glucose availability, since there were similar levels of glucose remaining in the media between treatments, suggesting similar glucose uptake (Figure 8b). In addition, after glycogen depletion and incubation with gluconeogenic substrates, glucose production via gluconeogenesis was unchanged between treatments (LFLS: 10.3 ± 0.58, LFHS 11.0 ± 0.50, HFHS: 11.9 ± 0.75 μM; *p* > .05). Insulin signaling, which inhibits gluconeogenesis, was significantly increased during acute stimulation with 100 nM insulin; however, this response did not differ between treatments (Figure 8c).

### 3.7 RNA sequencing

Given the limited differences between LFLS‐ and LFHS‐treated cells, we assessed changes in gene expression compared to LFLS‐treated cells only. Comparison of gene expression measured by RNA sequencing analysis showed that treatment of hepatocytes with HFHS media induced a limited number of significant gene changes compared to LFLS‐treated cells (eight upregulated and five downregulated, FDR *q* < 0.1); LFHS conditions were also able to induce gene changes compared to baseline, but not as many (one upregulated, (LINC00261 (ENSG00000259974)), FDR *q* < 0.1).

Subsequent functional analysis using gene set enrichment methods, revealed that only HFHS‐induced gene changes were significantly enriched in any Reactome functional pathways. There were nine metabolic pathways changed in the HFHS condition compared to the LFLS condition (Table 2). These were all enriched in genes down regulated after the HFHS treatment, with the exception of cholesterol biosynthesis, which was significantly enriched in genes overexpressed in HFHS‐treated cells. In addition, 28 cell cycle‐related pathways, 15 RNA processing‐stability pathways, 6 translation regulation pathways, 5 transforming growth factor beta pathways, and 5 viral machinery pathways were significantly enriched in genes downregulated in the HFHS treatment compared to the LFLS treatment (Table 3).

**TABLE 2:** Gene Set Enrichment Analysis (GSEA) of changes induced by HFHS versus LFLS conditions

REACTOME pathways	HFHS versus LFLS		
	SIZE	NES	FDR q‐val^a^
Metabolism			
Cholesterol biosynthesis	19	2.39	<0.001
Regulation of hypoxia inducible factor hif by oxygen	17	−1.86	0.005
Metabolism of proteins	338	−1.86	0.005
Respiratory electron transport atp synthesis by chemiosmotic coupling and heat production by uncoupling proteins	67	−1.86	0.005
RESPIRATORY electron transport	55	−1.87	0.005
Regulation of glucokinase by glucokinase regulatory protein	23	−1.73	0.016
Amino acid and oligopeptide slc transporters	28	−1.60	0.044
Amino acid transport across the plasma membrane	22	−1.60	0.044
Glucose transport	28	−1.60	0.044

^a^ Gene Set Enrichment Analysis by GSEA v3.0 on all metabolism genes ranked by the significance of fold change (http://software.broadinstitute.org/gsea). Only pathways significant at FDR‐adjusted *q* < 0.05 in HFHS compared to LFLS conditions are shown. NES, Normalized Enrichment Score; FDR, False Discovery Rate (Subramanian et al., 2005)

**TABLE 3:** Gene Set Enrichment Analysis (GSEA) of changes induced by HFHS versus LFLS conditions

REACTOME pathways	HFHS versus LFLS		
	SIZE	NES	FDR q‐val^a^
Metabolism			
Cholesterol biosynthesis	19	2.39	0.000
Regulation of hypoxia inducible factor hif by oxygen	17	−1.86	0.005
Metabolism of proteins	338	−1.86	0.005
Respiratory electron transport atp synthesis by chemiosmotic coupling and heat production by uncoupling proteins	67	−1.86	0.005
Respiratory electron transport	55	−1.87	0.005
Regulation of glucokinase by glucokinase regulatory protein	23	−1.73	0.016
Amino acid and oligopeptide slc transporters	28	−1.60	0.044
Amino acid transport across the plasma membrane	22	−1.60	0.044
Glucose transport	28	−1.60	0.044
Cell cycle			
Mitotic M M G1 phases	128	−2.21	0.000
DNA replication	144	−2.13	0.000
Influenza viral RNA transcription and replication	95	−2.44	0.000
Cell cycle checkpoints	92	−2.06	0.000
Mitotic prometaphase	63	−2.04	0.001
G1 S transition	84	−2.04	0.001
Mitotic G1 G1 S phases	101	−2.03	0.001
Regulation of mitotic cell cycle	67	−1.99	0.001
APC C CDC20‐mediated degradation of mitotic proteins	57	−1.99	0.001
ORC1 removal from chromatin	56	−1.98	0.001
Cell cycle mitotic	237	−1.95	0.002
Assembly of the prereplicative complex	54	−1.92	0.003
G0 and early G1	18	−1.86	0.005
APC CDC20‐mediated degradation of NEK2A	20	−1.88	0.005
Activation of the prereplicative complex	21	−1.86	0.005
APC C CDC20‐mediated degradation of cyclin B	18	−1.87	0.005
G2 M checkpoints	33	−1.86	0.005
Inhibition of the proteolytic activity of APC C required for the onset of anaphase by mitotic spindle checkpoint components	17	−1.87	0.005
M G1 transition	61	−1.87	0.005
S phase	87	−1.83	0.007
Cell cycle	286	−1.82	0.007
Synthesis of DNA	74	−1.82	0.008
Activation of ATR in response to replication stress	27	−1.77	0.011
APC C CDH1‐mediated degradation of CDC20 and other APC C CDH1‐targeted proteins in late mitosis early G1	56	−1.72	0.017
Cyclin E‐associated events during G1 S transition	52	−1.70	0.020
E2F‐mediated regulation of DNA replication	24	−1.70	0.020
CDT1 association with the CDC6 ORC origin complex	46	−1.65	0.033
Autodegradation of CDH1 by CDH1 APC C	49	−1.62	0.040
RNA processing‐stability			
Deadenylation‐dependent MRNA decay	39	−2.35	0.000
Metabolism of RNA	228	−2.53	0.000
Nonsense‐mediated decay enhanced by the exon junction complex	100	−2.60	0.000
Metabolism of MRNA	189	−2.60	0.000
Cytosolic tRNA aminoacylation	20	−1.91	0.004
RNA processing‐stability			
Deadenylation of MRNA	16	−1.89	0.005
Regulation of MRNA stability by proteins that bind Au‐rich elements	69	−1.83	0.007
Destabilization of MRNA by tristetraprolin TTP	15	−1.80	0.008
Destabilization of MRNA by BRF1	15	−1.74	0.015
Metabolism of noncoding RNA	42	−1.72	0.018
Processing of capped intron containing pre‐mRNA	117	−1.69	0.023
Transport of mature transcript to cytoplasm	47	−1.66	0.029
tRNA aminoacylation	34	−1.64	0.034
mRNA processing	134	−1.62	0.038
Transport of mature mRNA derived from an intronless transcript	29	−1.60	0.043
Translation regulation			
SRP‐dependent cotranslational protein targeting to membrane	103	−2.42	0.000
3 UTR‐mediated translational regulation	102	−2.61	0.000
Activation of the mRNA upon binding of the cap binding complex and EIFS and subsequent binding to 43S	53	−2.30	0.000
Translation	138	−2.42	0.000
Formation of the ternary complex and subsequently the 43S complex	46	−2.24	0.000
Peptide chain elongation	83	−2.65	0.000
TGfB signalling			
Downregulation of SMAD2 3 SMAD4 transcriptional activity	18	−1.81	0.008
SMAD2 SMAD3 SMAD4 heterotrimer regulates transcription	23	−1.75	0.014
Transcriptional activity of SMAD2 SMAD3 SMAD4 heterotrimer	34	−1.74	0.014
Signaling by TGF BETA receptor complex	54	−1.65	0.033
Downregulation of TGF BETA receptor signaling	20	−1.59	0.047
Viral machinery			
Influenza life cycle	125	−2.45	0.000
Interactions of VPR with host cellular proteins	28	−1.94	0.002
Host interactions of HIV factors	97	−1.83	0.007
Transport of ribonucleoproteins into the host nucleus	24	−1.81	0.008
NEP NS2 interacts with the cellular export machinery	24	−1.80	0.008
Miscellaneous			
Activation of genes by ATF4	20	−1.87	0.005
NOTCH1 intracellular domain regulates transcription	38	−1.79	0.010
Phosphorylation of the APC C	16	−1.73	0.015
Signaling by NOTCH1	53	−1.64	0.034
NETRIN1 signaling	22	−1.63	0.038
Cell death signalling via NRAGE NRIF and NADE	39	−1.61	0.044

^a^ Gene Set Enrichment Analysis by GSEA v3.0 on all genes ranked by the significance of fold change (http://software.broadinstitute.org/gsea). Only pathways significant at FDR‐adjusted *q* < 0.05 in HFHS compared to LFLS conditions are shown. NES, Normalized Enrichment Score; FDR, False Discovery Rate.

## 4 DISCUSSION

The purpose of this study was to create an in vitro model of TAG accumulation, with macrovesicular steatosis in Huh7 cells, by using nutritional substrates in physiological amounts/ratios, and investigate intracellular metabolism. To do this, we used three conditions that had a focus on sugar content; two were lower in FA (LFLS and LFHS) and one that was high in fat and sugar (HFHS). Overall, compared to LFLS and LFHS, HFHS media induced a number of metabolic changes relevant to NAFLD, including TAG accumulation and a LD pattern similar to isolated steatotic primary human hepatocytes. Previous cellular models that have induced steatosis have used between 100 and 2000 μM of either palmitate, oleate, or a combination of both, alongside sugars (5.5–25 mM glucose alone or with fructose), while others have used combinations of lactate (20 mM), pyruvate (2 mM), and octanate (4 mM) (Lyall et al., 2018), typically as a single dose over 24–48 hr (Chavez‐Tapia et al., 2012; Cui et al., 2010; Kostrzewski et al., 2017; Zhao et al., 2016). However, to replicate what the liver would be exposed to in vivo, we used a physiological ratio of four FAs (Hodson, Skeaff, & Fielding, 2008), including the two essential FAs, along with a mixture of sugars and HS. Rather than glucose alone, we used a low fat (200 μM) condition as a control in combination with 11 mM glucose; this level of glucose is used in primary human hepatocyte culture (Green et al., 2017) and was found to be optimal to replicate a fed/fasted cycle when media changes were carried out every 48 hr, similar to a recent 3D spheroid model of steatosis, which used repeated media changes for up to 21 days (Kozyra et al., 2018).

Although other in vitro NAFLD models have reported intracellular lipid accumulation, they have not characterized LD pattern (Chavez‐Tapia et al., 2012; Cui et al., 2010; Kostrzewski et al., 2017; Kozyra et al., 2018; Lyall et al., 2018; Zhao et al., 2016). Previous studies that had specifically set out to create large LDs (Nativ et al., 2013; Pawella et al., 2014; Yarmush et al., 2016) also utilized multiple doses of treatment media over a prolonged time period, similar to our protocol. Nativ *et al*. reported that treating primary rat hepatocytes and with 4 mM oleic and linoleic acid over 6 days successfully induced macrovesicular steatosis (LDs > 350 μm^2^) (Nativ et al., 2013). Long‐term culture (up to 40 days) of Huh7 cells with a combination of DMSO and a preadipocyte–adipocyte differentiation medium increased LD size and the expression of perilipin 1 (PLIN1), a LD protein associated with a macrovesicular pattern (Pawella et al., 2014). Although PLIN1 expression was unchanged in the present study, we found that the HFHS‐treated cells displayed a LD pattern similar to that seen in primary human hepatocytes isolated from fatty liver tissue. In addition, the quantity of intracellular TAG placed HFHS‐treated cells in the “NAFL2” grade of steatosis based on previously defined cut‐offs (220–465.5 nmol/mg of protein) (Chiappini et al., 2017; Peng et al., 2015).

One contributor to the intrahepatocellular TAG pool is DNL (Diraison et al., 2003; Donnelly et al., 2005). However, we found no difference between the LFLS‐ and LFHS‐treated cells in intracellular DNL‐derived FAs, while the contribution of DNL‐derived FAs in the HFHS‐treated cells was significantly reduced. Unsaturated FAs are known to downregulate DNL through liver X receptor and sterol regulatory element‐binding protein 1c (SREBP‐1c, encoded by the *SREBF1* gene) (Dentin, Hedrick, Xie, Yates, & Montminy, 2008; Hannah, Ou, Luong, Goldstein, & Brown, 2001; Ye & DeBose‐Boyd, 2011), while increasing levels of exogenous palmitate have been shown to downregulate DNL in human adipocytes (Collins et al., 2011); higher exogenous FA levels in HFHS media may therefore explain this result. Alternatively, in HFHS‐treated cells, DNL‐derived FAs may have been partitioned toward oxidation, or metabolized to FAs that were not measured, meaning their synthesis was not captured in our results. In human NAFLD, DNL is increased in the presence of insulin resistance, where lipogenic and gluconeogenic pathways are upregulated (Schwarz, Linfoot, Dare, & Aghajanian, 2003). To isolate the effect of nutrient composition, we exposed cells to the same amount of insulin regardless of treatment (0.5 nM); it may be that we detected the early stages of intracellular TAG accumulation and to induce insulin resistance, a more targeted approach and prolonged exposure to higher amount of insulin is required. Indeed, repeated stimulation with 100 nM insulin every 24 hr was required to elicit an upregulation of lipogenic gene expression in primary human hepatocytes, although whether this resulted in a functional change is not known, since DNL was not measured (Kozyra et al., 2018).

In the present study, there were no changes in DNL in LFHS‐ compared to LFLS‐treated cells; however, there was an effect of fructose at the gene expression level, with *SREBF1*, a key regulator in DNL, being more highly expressed in the LFHS compared to the HFHS condition. The *SREBF1* targets *FADS1* and *FADS2* were also upregulated in LFHS‐treated cells. To date, in vitro literature regarding the lipogenic effects of fructose is conflicting (Gnocchi, Massimi, Alisi, Incerpi, & Bruscalupi, 2014; Hirahatake, Meissen, Fiehn, & Adams, 2011; Huang et al., 2011; Lanaspa et al., 2012), while human studies where a fructose intervention diet is fed in isocaloric amounts relative to a control diet, do not support fructose as a cause of liver fat accumulation (Chiu et al., 2014; Chung et al., 2014). Nonetheless, the present data supports one of the proposed pathways of fructose‐induced lipogenesis, which is to upregulate DNL genes; however, it may be that in association with exogenous FAs, the inhibitory effects of unsaturated FAs on *SREBF1* expression (Hannah et al., 2001; Shimano & Sato, 2017) balances any stimulatory effect that fructose has. Indeed, a stronger association between fat, particularly saturated fat, and hepatic TAG accumulation seen under HFHS conditions is in agreement with human in vivo dietary intervention data (Parry & Hodson, 2017).

Apart from the effect of FAs on lipogenic transcription factors under the HFHS condition, there was also a downregulation of glucose transport and glycolysis pathways compared to LFLS‐treated cells evident from RNA sequencing. This contrasts with the glucose disappearance data, which did not change between conditions, but is supported by a reduction in lactate, the end product of anaerobic glycolysis, under HFHS conditions. The fate of glucose in HFHS‐treated cells is not clear, since RNA sequencing also showed a downregulation of oxidative phosphorylation, suggesting that glucose was not utilised for this process. Although we did not measure glycogenesis, it is possible that glucose not disposed of in DNL or glycolysis was used for storage. Interestingly, disruption of glycogen synthesis in primary mouse hepatocytes increased lipogenesis and insulin resistance, implying reduced glycogen synthesis could be causal in the hepatic insulin resistance present in NAFLD (Dashti, Alaupovic, Knight‐Gibson, & Koren, 1987). If the HFHS cells were able to dispose of glucose via glycogen synthesis, this may in part explain why neither insulin signaling nor DNL were changed in HFHS‐treated cells.

Although impaired insulin signaling may be expected to manifest under HFHS conditions, the association between liver fat and insulin resistance is complex: not all people with increased liver fat are defined as insulin‐resistant (Pramfalk, Pavlides, et al., 2016). This suggests that insulin resistance is not the only physiological response to intrahepatocellular TAG accumulation and may develop over time or require the presence of additional pathologies (e.g., inflammation). Impaired insulin signaling may not have been observed in our model as the cells were cultured in HS, which we have shown improves insulin signaling in Huh7 cells (Gunn et al., 2017). Thus, cells were more insulin‐sensitive to start with and may have required a longer, or more dramatic, “insult” before the insulin signaling cascade was affected. In addition, FA profiling showed that the HFHS‐treated cells had more long‐chain FAs in their PLs, while maintaining palmitate at a similar level to the low‐fat treated cells, suggesting a phenotype that may be less prone to insulin resistance, if palmitate is a causal factor in insulin resistance. Indeed, the primary cause of hepatic insulin resistance is much debated, which we have recently discussed (Hodson & Karpe, 2019).

The addition of FAs to the media in HFHS‐treated cells resulted in changes in the mobilization of FAs toward oxidation and secretion. Previous work has demonstrated that using HS and supplying exogenous FAs to culture media improves lipoprotein secretion in both HepG2 and Huh7 cells (Dashti et al., 1987; Ellsworth, Erickson, & Cooper, 1986; Gunn et al., 2017; Meex, Andreo, Sparks, & Fisher, 2011; Pramfalk, Larsson, Härdfeldt, Eriksson, & Parini, 2016; Steenbergen et al., 2013; Wu, Shang, Jiang, & Ginsberg, 1996). The use of HS over 30 days has been shown to replicate TAG and cholesterol lipoprotein profiles of human blood in Huh7 cells (Steenbergen et al., 2013); in the current model, we found that the combination of both HS and FAs not only resulted in cells secreting VLDL‐sized particles under all treatments, but the HFHS‐treated cells secreted the majority of TAG and cholesterol as VLDL particles after 7 days. Similarly, patients with NAFLD have been reported to have an overproduction of VLDL particles and an increase in particle size (Adiels et al., 2005; Adiels, Olofsson, Taskinen, & Borén, 2008); although a plateau in secretion may occur with increasing TAG accumulation (Fabbrini et al., 2008; Higuchi et al., 2011), suggesting the secretion capacity of HFHS‐treated cells was not yet overloaded.

To date, there is conflicting literature regarding whether energy production from ketogenesis and oxidation are changed in NAFLD (Bugianesi et al., 2005; Croci et al., 2013; Kotronen et al., 2009; Perla, Prelati, Lavorato, Visicchio, & Anania, 2017; Sanyal et al., 2001). The present data show that both pathways were upregulated in the HFHS condition, despite RNA‐sequencing data suggesting a downregulation of respiratory electron transport in high fat‐treated cells. It has been hypothesized that in the early stages of steatosis there is an increase in mitochondrial activity to dispose of excess FA, before microsomal oxidation and ROS increase and mitochondrial activity decreases (Koliaki et al., 2015; Peng et al., 2018). Our results are to a degree, in line with this hypothesis and although we appear to capture the shift away from mitochondrial oxidation, with changes detectable at a transcriptional level, we did not detect differences at the metabolic level, as supported by the lack of elevation in ROS under HFHS conditions. Thus, it is plausible that to detect increases in microsomal oxidation and ROS and a decrease in mitochondrial FA oxidation, a longer period of exposure to the HFHS culture media was required. The inclusion of linoleate in the culture media may also have impacted ROS production, since both oleate and linoleate have been shown to protect cells from palmitate‐induced oxidative stress and inflammation (Maruyama, Takahashi, Sekimoto, Shimada, & Yokosuka, 2014; Sommerfeld, Reinehr, & Haussinger, 2015). Interestingly, regulation of hypoxia inducible factor (HIF) by oxygen was noted to be downregulated from RNA‐sequencing data. Hypoxia is a hallmark of NAFLD (Anavi, Madar, & Tirosh, 2017) and the HIF target hypoxia‐inducible lipid droplet‐associated protein (HILPDA) gene expression is upregulated during LD growth (Breher‐Esch et al., 2018; Sahini & Borlak, 2016), suggesting low oxygen availability in steatosis is key in reducing FA oxidation and mediating LD growth through HILPDA.

Aside from the change in metabolic pathways identified from RNA sequencing, there were a large number of pathways relating to cell cycle, RNA processing and stability and protein translation regulation that were downregulated in HFHS‐treated compared to LFLS‐treated cells. An overall downregulation of cellular proliferation and production of cellular machinery through translation and transcription may suggest that HFHS‐treated cells were becoming senescent; senescence has been noted in NAFLD and is predictive of disease progression (Aravinthan et al., 2013). Of relevance in a high‐fat environment is a link between sphingolipid synthesis and cellular senescence, since palmitate is required for sphingolipid synthesis. In the present results under HFHS conditions, the cells were efficient in further metabolizing or partitioning palmitate away from storage: despite being exposed to a fourfold higher media FA concentration, HFHS‐treated cells had a significantly lower proportion of intracellular palmitate, compared to higher levels of oleate, linoleate, and α‐linolenate, which could be indicative of palmitate partitioning toward sphingolipid synthesis in HFHS cells. Indeed, previous work has shown an elevation of some sphingolipid species after high fat‐induced steatosis in a 3D spheroid model (Kozyra et al., 2018), supporting an association between steatosis development and cellular senescence.

There are limitations to our work. We used the Huh7 cell line, which does not fully recapitulate in vivo hepatocyte function. Although these cells secrete albumin (Jeon & Kim, 2011), due to being hepatoma derived, they show characteristics of dedifferentiation from mature hepatocytes: Huh7 cells express tumor markers and have limited expression of CYP enzymes, hepatic transcription factors, and nuclear receptors (Godoy et al., 2013). However, the cells do express key nuclear receptors (i.e., liver and farnesoid X receptor; LXR and FXR), transcription factors (i.e., SREBP1‐c and ChREBP), enzymes, and functional capability relevant to lipid metabolism, which are demonstrated in the current study. Moreover, we and others have found improved differentiation and metabolic function in hepatoma cells in response to culture in human serum (Gunn et al., 2017; Steenbergen et al., 2013). Indeed, in a comprehensive investigation, Huh7.5 cells cultured in adult human serum for 15 days (vs. 14 days in our study) showed restoration of key morphological and metabolic features of normal liver cells (Steenbergen et al., 2018).

In addition, although we compared lipid droplet pattern with primary hepatocytes, we were unable to replicate all work in primary cells as we found high variability in viability when isolating them from tissue, particularly fatty liver tissue. In addition, LDs in isolated primary cells were smaller than those seen in histology samples from the tissue hepatocytes were taken from (Figure 3). This suggests that the 3D architecture of the liver tissue allowed large LDs to form, and once broken down, a single cell could no longer facilitate large LDs; to recapitulate what is seen in tissue, use of 3D culture or organoids may be required (Kostrzewski et al., 2017; Kozyra et al., 2018). Finally, we focus or work on conditions with higher sugar, which meant we did not have a high fat, low sugar media in our comparisons; it would be of interest, given recent dietary trends, to do this comparison.

## 5 CONCLUSION

We have demonstrated that Huh7 cells exposed to repeated doses of a physiological, HFHS treatment developed a mixed macrovesicular steatotic LD pattern, similar to that found in isolated primary hepatocytes. These cells also display altered glucose metabolism, FA oxidation and lipoprotein secretion; RNA sequencing suggests that beyond metabolism, steatotic cells may be in a senescent state. Overall, this model displays several characteristics of early‐stage NAFLD in vivo thus providing the strong basis of a physiological model that can be further manipulated to investigate in vitro intracellular TAG development and/or regression.

## CONFLICT OF INTEREST

None.

## AUTHOR CONTRIBUTIONS

P.J.G.: study concept and design; acquisition of data; analysis and interpretation of data; drafting and revision of the manuscript, C.P.: analysis and interpretation of data, V.M.: acquisition of data, T.C.: acquisition of data, M.H.: acquisition of data, E.M.J.: acquisition of data, S.R.N.: acquisition of data, P.T‐R.: statistical analysis, R.F.M.: obtained funding, K.E.P.: acquisition of data, analysis and interpretation of data, M.H.T.: acquisition of data; analysis and interpretation of data, C.J.G.: study concept and design; data interpretation, revision of the manuscript, L.H.: study concept and design; data interpretation, obtained funding; drafting and revision of the manuscript. All authors read and approved the final manuscript.
